# Monomethyl Fumarate (MMF, Bafiertam) for the Treatment of Relapsing Forms of Multiple Sclerosis (MS)

**DOI:** 10.3390/neurolint13020022

**Published:** 2021-05-19

**Authors:** Amnon A. Berger, Emily R. Sottosanti, Ariel Winnick, Jonathan Izygon, Kevin Berardino, Elyse M. Cornett, Alan D. Kaye, Giustino Varrassi, Omar Viswanath, Ivan Urits

**Affiliations:** 1 Beth Israel Deaconess Medical Center, Department of Anesthesiology, Critical Care, and Pain Medicine, Harvard Medical School, Boston, MA 02115, USA; Emily.Sottosanti2@umassmed.edu; 2 Soroka University Medical Center and Faculty of Health Sciences, Ben Gurion University of the Negev, Beer-Sheva 8400100, Israel; ariel.winnick@gmail.com (A.W.); izygon@post.bgu.ac.il (J.I.); 3 School of Optometry, University of California, Berkeley, CA 94720, USA; 4 School of Medicine, Georgetown University, Washington, DC 20007, USA; kmb364@georgetown.edu; 5 Department of Anesthesiology, Louisiana State University Health Shreveport, Shreveport, LA 71103, USA; akaye@lsuhsc.edu (A.D.K.); viswanoy@gmail.com (O.V.); ivanurits@gmail.com (I.U.); 6 Paolo Procacci Foundation, Via Tacito 7, 00193 Roma, Italy; GIUVARR@GMAIL.COM; 7 Department of Anesthesiology, University of Arizona College of Medicine-Phoenix, Phoenix, AZ 85004, USA; 8 Valley Anesthesiology and Pain Consultants—Envision Physician Services, Phoenix, AZ 85001, USA; 9 Department of Anesthesiology, School of Medicine, Creighton University, Omaha, NE 68124, USA; 10 Southcoast Health, Southcoast Health Physician Group Pain Medicine, North Dartmouth, MA 02747, USA

**Keywords:** autoimmune, corticosteroids, disease-modifying agents, CNS plaques, disability

## Abstract

Multiple sclerosis (MS) is a prevalent neurologic autoimmune disorder affecting two million people worldwide. Symptoms include gait abnormalities, perception and sensory losses, cranial nerve pathologies, pain, cognitive dysfunction, and emotional aberrancies. Traditional therapy includes corticosteroids for the suppression of relapses and injectable interferons. Recently, several modern therapies—including antibody therapy and oral agents—were approved as disease-modifying agents. Monomethyl fumarate (MMF, Bafiertam) is a recent addition to the arsenal available in the fight against MS and appears to be well-tolerated, safe, and effective. In this paper, we review the evidence available regarding the use of monomethyl fumarate (Bafiertam) in the treatment of relapsing-remitting MS.

## 1. Introduction

Multiple sclerosis (MS) is a disease of the central nervous system (CNS) where the immune system attacks the myelin sheath, causing symptoms such as weakness, numbness, gait abnormalities, electric sensations, blurred vision, double vision for extended time periods, and bladder or bowel dysfunction [1]. Over 2 million people worldwide are affected by MS, and it may affect between 5 and 200 people per 100,000 [2]. MS commonly affects women more than men (2.3–3.5:1) and typically affects adults, with the average age of onset at 28–31 years of age [3,4].

Over the years, disease-modifying agents have been formulated to decrease the progression of the disease. Bafiertam (monomethyl fumarate, MMF) was FDA-approved in April 2020 as an oral treatment for relapsing forms of MS, including clinically isolated syndrome, relapsing-remitting disease and secondary progressive disease in adults [5]. It is not known if Bafiertam is safe and effective in children. The most common side effects associated with MMF include abdominal pain, flushing, nausea, and diarrhea.

Fumarates likely have neuroprotective and immunomodulatory properties [6]. Badiertam alters the nuclear factor (erythroid-derived 2)-like 2 (NFE2L2 or NRF2) transcription factor [5]. NRF2 regulates the expression of antioxidant proteins that can protect against oxidative damage and stress that can be triggered by inflammation and damage.

In this review, we will discuss detailed information about MS, current treatment options, and the clinical evidence for MMF as a treatment for MS.

This was a narrative review. In 2020–2021, we performed a comprehensive search for English-language studies related to monomethyl fumarate and Bafiertam as a treatment for MS. We searched the following databases: PubMed, Medline, SciHub, Cochrane Database of Systematic Reviews, and Google Scholar. We used the following combinations of keywords: autoimmune; corticosteroids; disease-modifying agents; CNS plaques; disabilities; multiple sclerosis; MMF; monomethyl fumarate: Bafiertam. We tried to include as many recent manuscripts as possible (within the last three years) but also included papers that were older than three years if they were particularly relevant to our topic. We also attempted to search for, use, and cite primary manuscripts whenever possible.

## 2. General Information about MS

### 2.1. Multiple Sclerosis Classifications

The first clinical episode of MS is a clinically isolated syndrome (CIS) [1,7]. It is characterized by a monophasic clinical episode with symptoms reflecting focal or multifocal inflammatory demyelination of the CNS [8]. The duration of symptoms should last no less than 24 h and should be in the absence of any signs of infection [8]. Symptoms can include painless diplopia due to internuclear ophthalmoplegia (or sixth nerve palsy), cerebellar syndrome, facial numbness, partial transverse myelitis (i.e., Lhermitte sign), urge incontinence, and most notably unilateral optic neuritis [1,9]. Symptoms develop within hours to days and can last for months.

Relapsing-remitting MS (RRMS) involves clearly defined attacks in the form of flares, relapses, or worsening of symptoms [7]. Symptoms are not known to progress between relapses. RRMS is often characterized as CNS dysfunctions separated in time and space. The dysfunction must occur on two separate occasions in different parts of the CNS for this classification to be diagnosed. In 85–90% of MS cases, it is the most common subtype [10].

Secondary progressive MS (SPMS) is considered the progression of an RRMS disease course. Although there are no established criteria to determine the onset of secondary progressive MS, it is considered to follow a gradually worsening disease course with minor remissions [7]. Since there are no established criteria nor sentinel event, the diagnosis of secondary progressive MS is usually made in retrospect [11]. Progression from relapsing-remitting to secondary progressive has been known to occur within 10–20 years of disease onset [12]. Secondary progressive MS may be further classified as active or non-active and with progression or without progression. An “active” state is defined as novel MRI activity and/or presence of relapse, whereas progression can be determined when objective worsening of disease is present over time.

Primary progressive MS (PPMS) is largely defined by patient history. Neither imaging nor exam findings may be used to distinguish it from relapse-remitting MS [13]. This form generally requires that symptoms progressively accumulate since the symptom onset, with only minor and temporary improvements or occasional plateaus. Primary progressive MS can present as a spinal cord syndrome in the form of an asymmetric spastic paresis, which gradually worsens with time [14]. PPMS less commonly presents with cerebellar ataxia and visual disturbances. Much like SPMS, primary progressive can be subdivided as active/inactive and progressive/nonprogressive.

### 2.2. Disease Progression

Each subtype of MS has a different progression of the disease. Most cases follow a relapsing-remitting course. As mentioned above, RRMS appears in the form of flares that present across time and space (within the CNS) [7]. PPMS presents with symptoms accumulating and worsening over time, which occasionally plateau with little to no improvement. Common MS symptoms include sensory loss in the limbs or one side of the face, unilateral visual loss, motor weakness which presents acutely or sub-acutely, diplopia, gait, and balance disturbances, and Lhermitte sign, which is described as an electric sensation along the back or limbs with flexion of the neck [15]. Vertigo, bladder issues, limb ataxia, transverse myelitis, and pain are also common presentations. Patients may have single or multiple symptoms, consistent with single or multiple lesions, respectively. Patients may also experience cortical syndromes such as aphasia, although this is uncommon.

### 2.3. Pathophysiology

Although there is no agreed-upon cause of MS to this day, the major pathologic mechanisms that seem to cause the clinical presentation of MS are inflammation, demyelination, and axonal degeneration [4,16,17]. One prevalent theory regarding MS pathogenesis involves an inflammatory immune-mediated disorder characterized by autoreactive lymphocytes and progresses to a predominant microglial activation and neurodegeneration [16,18]. These autoreactive lymphocytes are thought to start the pathogenic cascade, which culminates in demyelination, neuroaxonal degeneration, synaptic loss, oligodendrogliopathy, and eventually, astrogliosis and tissue loss [19]. Focal demyelinated plaques, along with inflammation and gliosis, are typical features of the neuropathology of MS [20]. These lesions are commonly located at the optic nerves, brainstem, cerebellum, and periventricular white matter [21]. Another prevalent theory is mitochondrial dysfunction and respiratory chain deficiency due to prolonged inflammation and chronic oxidative stress [22]. This creates an energy imbalance that can exacerbate neurodegeneration. In recent years, the MS spectrum encompasses a large range of abnormalities. This includes diffuse damage of normal-appearing white matter (NAWM) and normal-appearing gray matter (NAGM) on magnetic resonance imaging (MRI) [23]. These are both associated with the progressive loss of brain volume [23].

### 2.4. Risk Factors

Several environmental risk factors and exposures are associated with the development of MS. One of the strongest associations with MS is the Epstein–Barr virus (EBV) [24]. The presence of antibodies or seropositivity is consistently associated with MS in people of different races and ethnicities [25,26]. Strengthening this association, a nested case–control study demonstrated that adults with MS who were initially EBV-negative were shown to be antibody-positive before the onset of disease [27]. A meta-analysis of the risk of MS in EBV positive patients found that patients with a history of infectious mononucleosis are over twice that of the general population [28]. As mentioned above, ultraviolet radiation exposure and vitamin D are associated with decreased chances of developing MS [24]. Some of the strongest evidence for a causal relation of vitamin D status and MS is given by mendelian randomization studies, which have shown the association of genetic variants affecting serum vitamin D and MS risk [29]. However, these associations are not present in African Americans or Hispanics [30]. Cohort and case–control studies show an association between obesity and MS risk, where obesity in childhood and adolescence, but not adulthood, may be associated with subsequent risk of MS [31]. Finally, a dose-dependent association of cigarette smoke and MS has been found in a large case–control study and pooled analysis of other studies [32]. This may be due to lung irritation, which can trigger an inflammatory and autoimmune response, rather than tobacco use [33]. MS is also known to have genetic risk factors. This has been shown through the familial clustering of MS and the increased prevalence of MS in specific racial groups [34]. Genes at the HLA antigen locus seem to have the strongest effect on MS risk, with HLA-DRB1*1501 causing three times the risk of developing the disease [35]. HLA-A*02 is associated with reduced odds of developing MS [36]. Many genetic variants on varying chromosomes have been found to affect susceptibility to the disease over the years [37]. These variants are located on noncoding regions of the genome, leading researchers to believe they affect regulatory mechanisms [37]. Additionally, they have, in the large part, been localized near genes that regulate innate or adaptive immunity [38].

### 2.5. Diagnostic Criteria

A typical presentation of MS involves a young adult with one or more clinically distinct episodes of CNS dysfunction, such as optic neuritis, long tract signs, or brainstem/spinal cord syndrome, which are typically followed by partial resolution [39]. These symptoms develop and progress over the span of hours to days and, subsequently, depending on the type of MS, may remit over the following weeks to months [9]. Diagnosis of MS necessitates a clinical history that probes for prior attacks with symptoms and progression of symptoms characteristic of inflammatory demyelination in the CNS [40]. All patients must be assessed with an MRI unless contraindicated [41]. In cases where there is insufficient clinical and MRI evidence, but the presentation is typical to MS, confirmation of diagnosis may be accomplished through additional tests such as a lumbar puncture and visual evoked potentials. Additionally, physicians may use the McDonald criteria to help correctly diagnose the patient. The McDonald criteria are a collection of conditions that help the physician utilize the correct diagnostic protocol based on the patient’s clinical presentation [42]. Of note, if there is a lack of clinical evidence in favor of MS, these studies cannot be used to support optic nerve lesions.

## 3. Current Treatment Options

Corticosteroids have been used to treat MS since the 1950s and are still considered the standard treatment for acute exacerbations [43,44]. Treatment formulations include intramuscular and intravenous (IV) administration of ACTH and synthetic glucocorticoids (oral prednisone, oral dexamethasone, oral prednisolone, and oral/IV methylprednisolone) [45]. A Cochrane review demonstrated short-term high-dose IV methylprednisolone improved MS symptoms without significant side effects, which are mostly related to chronic corticosteroid use [46,47]. Overall, while the best evidence exists for high-dose IV methylprednisolone, there is no firm evidence to conclude that one agent, dose, or route of administration has a clear benefit over the others [45,48]. Corticosteroids work by suppressing inflammation, which improves motor functioning and shortens the recovery from acute attacks. While many potential adverse effects are attributed to long-term usage, minor and dose-related side effects can be seen with short courses as well (e.g., behavioral effects, sleep derangements, hypertension, diabetes, lipid derangements, among others). There is not sufficient evidence to suggest that long-term corticosteroid treatment delays the progression of MS. Thus, it is mainly reserved for acute exacerbations and in combination with long-term disease-modifying therapy [44].

Plasma exchange began being utilized in neuroimmunology in the 1980s for myasthenia gravis and subsequently for MS. This strategy is designed to remove circulating autoantibodies, cytokines, immune complexes, and other inflammatory mediators from plasma [44,49]. A randomized controlled trial demonstrated moderate or greater improvement in neurological disability in 42.1% of patients treated with plasma exchange compared with 5.9% of controls [50]. The best effects are typically seen when therapy is administered within 4–6 weeks after symptom onset, with therapeutic effects occurring after a minimum of three sessions. However, overall, there is still limited evidence regarding its efficacy. It is not recommended as a permanent disease-modifying therapy but can be considered as a treatment option on an individual basis for patients with severe relapses [44,51].

The approved “first-generation” self-injectable disease-modifying therapies include four interferon (IFN) beta preparations and glatiramer acetate (GA). IFN beta modulates the function of T-cells, B-cells, and reduces blood–brain barrier disruption while GA stimulates regulatory T cells [47,52]. However, their mechanisms are not fully understood. These options have comparable efficacy, reducing the development of new brain MRI lesions from 1 to 3 years and the clinical relapse rate by approximately one-third [47,53,54,55,56]. IFN beta interventions also slow the worsening of the Expanded Disability Status Scale (EDSS) in relapsing MS patients but have little effect on progressive MS patients [47,57,58,59]. Even with the advent of new therapies, IFN beta and GA remain first-line treatments due to favorable long-term safety profiles and little monitoring requirements [47].

Natalizumab is a humanized monoclonal antibody that targets cell adhesion molecules on the surface of monocytes and lymphocytes, preventing transmigration of these immune cells across the blood–brain barrier [47,60]. Natalizumab has shown significant efficacy against EDSS-measured disability, clinical relapses, and MRI-measures, which led to its FDA approval for relapsing MS in 2004 [47,61,62,63]. Natalizumab is generally well-tolerated, but it is associated with an increased rate of common infections such as pharyngitis. Furthermore, two natalizumab trial participants developed progressive multifocal leukoencephalopathy (PML) due to the disruption of normal immune surveillance [47,64,65,66]. This led to the temporary withdrawal of natalizumab, but it was reintroduced in 2006 with a risk mitigation and PML monitoring strategy (the TOUCH program) [47]. MRI or clinical evidence that suggests PML prompts natalizumab discontinuation and further investigation. Given this risk of PML, natalizumab is typically reserved for patients with “breakthrough” disease with one or more of the first-line treatments. However, in early aggressive MS, some specialists use natalizumab as first-line therapy for 1–2 years, followed by a transition to a different agent [47].

Teriflunomide, fingolimod, and dimethyl fumarate (DMF) are oral therapies approved for relapsing MS. Teriflunomide is FDA approved for relapsing MS and has shown efficacy in relapse rate and MRI disease activity. It is the active metabolite of leflunomide and acts as an inhibitor of dihydroorotate dehydrogenase, an enzyme involved in de novo pyrimidine synthesis of proliferating cells [47,67]. Teriflunomide is generally well tolerated at approved doses; however, common adverse effects include elevated liver transaminases (for which it carries a black box warning for potential hepatotoxicity), lymphopenia, nausea, hypertension, peripheral neuropathy, diarrhea, alopecia, and acute renal failure. Furthermore, teriflunomide is a teratogen and is excreted in breast milk and semen. It also has a prolonged half-life of 18–19 days due to enterohepatic recirculation and can take up to 2 years to be fully eliminated from the body after discontinuation, which is a potential concern in patients who may become pregnant [47,68]. In these patients, activated charcoal or cholestyramine can be used to accelerate elimination over an 11-day period [47,69]. As such, teriflunomide is a less suitable option in women of childbearing potential, patients with preexisting hepatic conditions, or those with a history of nonadherence to medications or monitoring [47].

Fingolimod is FDA-approved for relapsing MS and has shown superiority to placebo and intramuscular IFN beta-1a in measures of MRI disease activity and clinical relapse [47,70,71]. It acts as a sphingosine-1-phosphate (S1P) functional antagonist that interferes with the exit of lymph nodes by lymphocytes [47,72]. Trapping lymphocytes in the nodes makes them unavailable to enter the central nervous system (CNS) and create MS lesions. Fingolimod also affects neurons and supporting glia in the CNS that express S1P receptors [47,73]. Adverse effects are related to the effects of lymphopenia (e.g., risk of viral infections) and interactions with S1P receptors in other tissues (e.g., retina, cardiac, smooth muscle). Fingolimod is a reasonable first or second-line treatment for patients without cardiovascular risk factors and those with a high likelihood of adherence to consistent monitoring [47].

**Figure 1:**
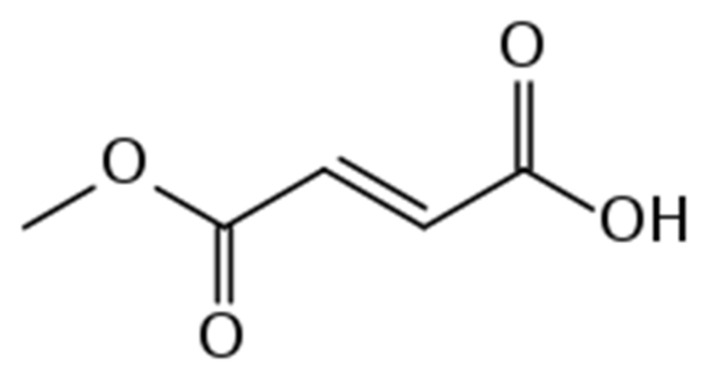
Organic structure of monnomethyl fumarate.

**Figure 2:**
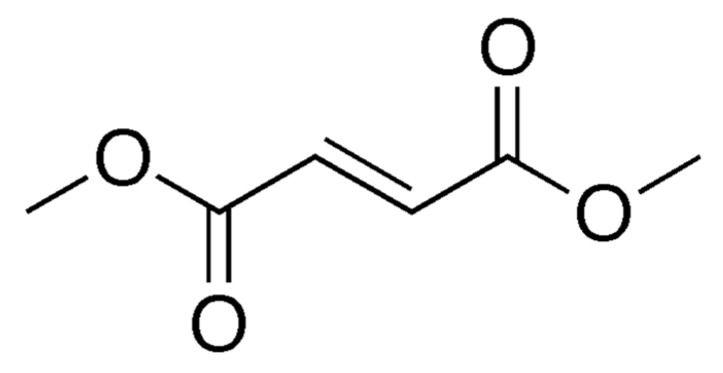
Organic structure of dimethyl fumarate.

DMF is also FDA-approved for relapsing MS. It is metabolized to monomethyl fumarate (MMF) and eliminated via respiration with little renal or hepatic excretion (see Figure 1 and Figure 2). Its mechanism is not entirely clear; however, it is known to work through activation of nuclear-related factor 2 (Nrf2) and nuclear factor-kappa beta, which reduce oxidative cell stress and inflammation, respectively [6,47,74]. DMF has demonstrated a significant reduction in relapse rate and MRI disease activity compared with placebo and has also outpaced GA on these measures, although it demonstrates no additional benefit on EDSS progression [47,75,76]. DMF’s safety profile is relatively favorable overall. However, roughly 30% of individuals will experience self-limited flushing or gastrointestinal (GI) side effects (e.g., nausea, diarrhea, abdominal pain). Due to an average lymphocyte reduction of 30%, regular monitoring of complete blood cell count is recommended to prevent opportunistic infections such as PML [47].

Mitoxantrone is a general immunosuppressant approved for rapidly worsening MS and secondary progressive MS [47,77]. At standard dosing, it is limited to two years of use due to its risk of dose-related cardiomyopathy and treatment-related acute leukemia [47,78]. Other emerging therapies include laquinimod (an oral agent), alemtuzumab, ocrelizumab, and daclizumab (immune therapy agents). These therapies have shown promise in clinical trials and could play a role as combination or monotherapies in the future [47]. 

Overall, while there have been remarkable advances in the treatment options for MS, there is a lack of comparative data for clinical evidence of these options, hindering the evaluation of their therapeutic value [47,79]. Establishing an effective treatment plan often requires extensive coordination between the patient and provider to develop a personalized strategy that maximizes effectiveness and limits adverse events based on individual risks [47]. 

## 4. Monomethyl Fumarate (MMF, Bafiertam)

MMF (trade name BAFIERTAM^TM^) is the active metabolite of DMF that was approved by the U.S. Food and Drug Administration (FDA) in April 2020 for the treatment of relapsing MS in adults (including active secondary progressive disease, relapsing-remitting disease, and clinically isolated syndrome) [5]. It is administered in delayed-release 95 mg oral capsules. The starting dose is 95 mg twice per day for seven days, followed by a maintenance dose of 190 mg twice per day [5].

### 4.1. Pharmacology of MMF

MMF is the active metabolite of DMF, which is believed to exert its effect through activation of Nrf2 and nuclear factor-kappa beta to reduce oxidative cell stress and inflammation [6,47,74]. Nrf2 activation by MMF has demonstrated cytoprotection in human astrocytes via the OSGIN1 transcriptional target [80]. Additionally, MMF decreases the expression of vascular cell adhesion molecules, thereby reducing the adhesion and transendothelial migration of monocytes across an inflamed human blood–brain barrier (BBB) [81]. MMF can also modulate the immune response by impairing the maturation of dendritic cells and their activation of T cells [82]. Furthermore, activation of the Nrf2 pathway by monomethyl fumarate has a neuroprotective effect on ischemia-reperfusion in rats [83,84]. However, as with DMF, the precise mechanism of action remains unknown [5,47].

Following 190 mg oral administration of MMF, its median T_max_ is 4.03 h with a bioequivalent C_max_ (peak plasma concentration) and AUC (overall exposure) to that following 240 mg oral administration of DMF. While a high-calorie, high-fat meal does not significantly affect the AUC of MMF, it decreases its C_max_ by 20% and prolongs the median T_max_ from 4 to 11 h [5]. In healthy subjects, its apparent volume of distribution varies from 53 to 73 L. Overall, 27–45% is bound by human plasma proteins regardless of serum concentration. Metabolism occurs through the tricarboxylic acid cycle without the involvement of cytochrome P450 (CYP450) enzymes, thus minimizing interactions with other drugs. Major metabolites of MMF include citric acid, fumaric acid, and glucose. Based on studies with DMF, exhalation of CO_2_ accounts for approximately 60% of excretion, with renal (16%) and fecal (1%) routes of elimination contributing minor roles as well [5,47]. The half-life of MMF is approximately 30 min, leaving no serum levels present in the majority of individuals under fasting conditions 24 h after a 190 mg dose. No dosage adjustments are recommended for differences in age, gender, or body weight [5].

### 4.2. Side Effects/Adverse Events of MMF

The side effects associated with MMF are possible side effects and common side effects. Common side effects include flushing, redness, itching, or rash [5]. They also include nausea, vomiting, diarrhea, stomach pain, or indigestion. Of these side effects, flushing and stomach problems are the most commonly occurring reactions, usually at the beginning of the treatment and should decrease over time. The possible side effects include allergic reaction in the form of welts, hives, swelling of the face, lips, mouth or tongue, or difficulty breathing [5]. PML is another possible side effect that is a rare brain infection which can lead to death or severe disability over several weeks or months. Symptoms of PML include weakness on one side of the body that gets worse, vision problems, confusion, clumsiness in the arms or legs, changes in memory or thinking, and personality changes. Herpes zoster infection (shingles) is another central nervous system infection that can occur as a side effect. Patients should also be aware of decreases in white blood cell count and should have their white blood cell count monitored before starting treatment and every sixmonths after starting treatment [5]. Liver problems are also a concern. MMF may cause liver problems that can lead to liver failure, liver transplant, or death. Liver function should be monitored by a physician before starting treatment, and patients should note these signs of liver problems during treatment, including severe tiredness, loss of appetite, pain on the right side of stomach, dark brown colored urine, and yellowing of the skin or whites of the eyes [5].

## 5. Important Clinical Studies Involving MMF and MS

Data on the efficacy of Bafiertam™ (MMF) in patients with relapsing forms of multiple sclerosis come from the results of several clinical trials, to date including DEFINE, CONFIRM, their ongoing extension ENDORSE, and retrospective analyses of pooled trial data [75,76,85,86,87,88,89,90,91,92,93,94]. It is important to note that monomethyl fumarate (MMF; Bafiertam™) is the sole active metabolite detectable in plasma of dimethyl fumarate (DMF; Tecfidera^®^) and diroximel fumarate (DRF; Vumerity^®^). For this reason, efficacy data for monomethyl MMF are based on bioequivalence with dimethyl fumarate (DMF) preapproval clinical trial data for DMF, and post-market monitoring of patients treated with DMF.

Phase I studies compared the safety profile of DMF and DRF, both prodrugs of MMF, and recent head-to-head phase I trials have more directly investigated the tolerability of MMF versus DMF in healthy volunteers (see also above, Safety, Adverse Events) [95,96]. In an early pilot study, the effects of fumaric acid esters (FAEs) were investigated in 10 patients with a diagnosis of definite relapsing active multiple sclerosis (RRMS) [97]. The FAE preparations administered to MS patients in this study contained high amounts of DMF and were originally approved in Germany for the treatment of severe plaque psoriasis (Fumaderm^®^; Fumapharm, Muri, Switzerland). Enrolled patients had active lesions on MRI and showed reductions in the mean number and total volume of gadolinium enhancing lesions (GdE) on T1 MRI after 18 weeks of treatment with 720 mg oral FAE [97]. A more recent phase I comparative study of monomethyl fumarate (MMF) evaluated the side effects and safety profile of MMF in healthy volunteers [95]. In this study, 210 healthy volunteers (159 female) were administered equal molar weights of MMF (190 mg) and DMF (240 mg) twice daily over a 5 week treatment period. The gastrointestinal tolerability profile of MMF, assessed by the Modified Overall Gastrointestinal Symptom Scale (MOGISS), showed no statistically significant differences in terms of primary study endpoints, i.e., area under the curve (AUC), over the 5 week treatment period. The study authors noted statistically significant differences in mean worst severity MOGISS scores overall, with lower scores for vomiting and diarrhea with Bafiertam™.

For phase II studies of dimethyl fumarate (DMF), an early phase IIb multicenter, randomized, double-blind, placebo-controlled trial of oral DMF investigated safety and efficacy of 120 mg QD, 240 mg TID, and 240 mg TID doses in patients with RRMS compared to placebo [86]. It was found that the 240 mg TID dose reduced the mean number of GdE lesions by 69% over a 12-week period (primary endpoint), reduced the number of new or enlarging T2 MRI hyperintense lesions, number of new T1-hypointense lesions, and reduced annual relapse rate (ARR) by 32%. The authors also noted that the study was not adequately powered for relapse endpoints.

Two large phase III trials of DMF were conducted in 2012, one comparing DMF to placebo and the other comparing DMF to glatiramer as active comparator [75,76]. The first multicenter, randomized, double-blind, placebo-controlled trial was Determination of the Efficacy and Safety of Oral Fumarate in Relapsing-Remitting MS (DEFINE) [76]. This study investigated the use of oral DMF (BG-12) in RRMS patients (*n* = 1234) by giving them 240 mg of DMF twice daily, 240 mg of DMF thrice daily, or placebo. A significantly lower estimated proportion of relapse was found in the DMF groups, 27% in patients taking BID DMF, and 26% in patients taking TID DMF, versus 46% with placebo. The annualized relapse rate (ARR) at two years was 0.17 with BID, 0.19 with TID, and 0.36 with placebo (annualized relapse rate defined by the total number of relapses divided by the number of patient years). The rate of disability progression, as measured by the Expanded Disability Status Scale (EDSS), was 16% with BID, 18% with TID, and 27% with placebo. Additionally, there were significant reductions in the number of Gd+ T2 MRI hyperintensities with both DMF regimens compared to placebo.

The second randomized, multicenter, double-blind trial to evaluate the efficacy of DMF was Comparator and an Oral Fumarate in Relapsing–Remitting Multiple Sclerosis (CONFIRM) [75]. This study investigated the use of oral DMF (BG-12) in RRMS patients (*n* = 1417) by giving them 240 mg of DMF twice daily, 240 mg of DMF thrice daily, 20 mg of glatiramer acetate (GA) subcutaneously daily, or placebo. The annualized relapse rate at 2 years was 0.22 with BID DMF, 0.20 with TID DMF, 0.29 with glatiramer acetate, and 0.40 with placebo. Significant reductions were found in the number of new or enlarging T2 hyperintensities, and reductions in new T1 hypointensities with both DMF and GA. No significant reductions in disability progression were found between the DMF regimens and GA. Overall, the DEFINE and COMPARE trials found that treatment regimens of DMF significantly reduced the proportion of relapses, disability progression, and the number of MRI lesions in RRMS patients compared to placebo.

Additional information about the efficacy of DMF comes from retrospective data analyses from the DEFINE, CONFIRM, and now ENDORSE clinical trials, plus analyses of real-world comparative clinical data from several large MS clinics and national registries [88,89,90,91,92,93,98,99,100,101,102,103,104]. In further analyses of the randomized controlled clinical trials, retrospective studies consider subgroups of RRMS patients or combined patient data from multiple studies. For example, analysis of MRI data from a small group of patients in the abovementioned phase IIb trial showed a significantly lower percentage of evolution of Gd+ lesions to T1 hypointense lesions [98]. Patients who had received 240 mg of BG-12 (DMF) thrice daily (TID, *n* = 18) versus placebo (*n* = 38) showed reduced lesion evolution, even after adjusting regression models for disease duration and relapse activity. In another analysis of the same phase IIb trial, baseline characteristics and demographic data of 108 patients (240 mg DMF TID, *n* = 54; placebo, *n* = 54) showed reductions in the number of new Gd+ lesions among numerous subgroups [88]. Subgroups with significant reductions included EDSS score ≤ 2.5, EDSS > 2.5, no Gd+ lesions, ≥ 1 Gd+ lesion, age < 40 years, age ≥ 40 years, female patients, disease duration ≤ 6 years, and disease duration > 6 years. It was noted that only the male subgroup showed no significant reductions in the number of new Gd+ lesions. In subgroup analyses of the DEFINE clinical trial data, it was found that for all subgroups, DMF BID and TID reduced the proportion of patients who relapsed, and the annualized relapse rate (ARR), compared to placebo [92]. Further analyses of the CONFIRM trial data revealed that health-related quality of life (HRQoL) measures had improved significantly at two years from baseline scores [90]. These HRQoL measures included the Physical Component Summary (PCS), Mental Component Summary (MCS), and Short Form-36 (SF-36). Statistically significant improvements were noted for both DMF BID and glatiramer acetate (GA) compared to placebo, with a trend towards improvement with DMF TID. Similar results were also shown for patient-perceived health status measures such as PCS, MCS, and SF-36 with an analysis of the DEFINE trial data [91]. In post hoc analyses of pooled data from the DEFINE and CONFIRM trials, newly diagnosed patients (*n* = 678) naïve to MS disease-modifying therapy showed statistically significant clinical and neuroradiological outcomes at 2 years [89]. For patients diagnosed with RRMS within 1 year of study entry, 240 mg of DMF BID (*n* = 221) reduced the ARR by 56%, and 240 mg of DMF TID (*n* = 234) reduced the ARR by 60%, compared to placebo. Another subset of patients with available MRI data (*n* = 308) analyzed for neuroradiological progression of disease showed relative reductions in adjusted mean number of new or enlarging T2-hyperintense lesions at 2 years, 80% with 240 mg of DMF BID (*n* = 221), and 81% with 240 mg of DMF TID (*n* = 234), compared to placebo.

Several real-world clinical studies and retrospective analyses of patient data have provided additional efficacy information for DMF treatment in MS patients. These studies have considered MS patient sub-populations and/or data for active comparators such as fingolimod (FTY) and teriflunomide (TRF) [99,100,101,102,103,104]. In a retrospective chart review of RRMS patient (*n* = 390) initiating DMF treatment at US tertiary clinics, the efficacy of DMF was found not to differ among White Americans, African Americans, and Hispanic Americans [99]. A study of real-world efficacy of DMF in RRMS patients at a large academic MS center found that DMF (*n* = 458) and fingolimod (FTY) (*n* = 317) had comparable clinical efficacy [100]. MRI activity and rates of discontinuation between patients taking DMF and FTY in this study were also comparable. A real-world study of 119 patients (59.7% female) from the national MS registry of Kuwait evaluated MS patients taking 240 mg DMF BID for at least six months (mean duration 20.5 ± 9.5 months) [101]. In this study, the proportion of relapse-free patients increased significantly from 51.2% to 89.9%, and the proportion of patients with MRI activity decreased significantly from 61.1% to 15.1%. Analysis of combined patient data from two US academic MS centers showed the proportion of relapse was similar for those prescribed DMF (*n* = 737) compared to FTY (*n* = 535) [102]. Patients taking DMF in this study were more likely to discontinue treatment (*n* = 326) than patients taking FTY (*n* = 186). The study authors cited intolerability as the likely main factor responsible for this difference. Retrospective analysis of data from six MS centers in Italy (*n* = 456) showed an ARR reduction of 75% compared to baseline ARR during DMF treatment, and DMF discontinuation significantly associated with a higher baseline EDSS [104]. Finally, a recent comparative trial provided Class III evidence for similar clinical effectiveness of DMF and teriflunomide (TRF) in RRMS patients at 2 years post-initiation [103]. In this study, patients taking DMF (*n* = 1057) and patients taking TRF (*n* = 713) had similar relapse rates and disability progression, but the proportion of patients with at least one new T2 lesion was significantly lower with DMF (60.8%) compared to TRF (72.2%). In the context of these and other trials, a large database study also raised the issue of the lack of comparative evidence and data on clinical effectiveness for the use of DMF in MS patients in the post-approval period, citing a lack of direct comparison, from analysis of 16 trials of MS disease-modifying drugs compared to placebo (11) and to interferon-β-1a (5) [79].

Interim analysis of ENDORSE, the ongoing 12-year extension of DEFINE and CONFRIM, now provides data on the extended use of DMF in RRMS patients for up to 5 years [87]. In patients continuing to take 240 mg of DMF BID (BID/BID), cumulative ARR during years 0–5 was 0.163. For patients taking glatiramer acetate (GA) in CONFIRM (GA/BID), cumulative ARR during years 0–5 was 0.199. Detailed analysis of early ARRs was also reported for DMF, compared to both placebo and GA. The study authors reported consistently low clinical MRI activity with analysis at 5 years, i.e., in the succeeding 3 years after 2 years of DEFINE and CONFIRM. The more recent analyses of ENDORSE reported sustained efficacy of DMF for up to 11 years, i.e., in the succeeding 9 years after DEFINE and CONFIRM [93]. Over the approximately 9 years of ENDORSE, 47% of patients initially randomized to placebo who switched to DMF were relapse free, as were 53% of patients randomized to DMF who continued taking DMF. The authors also noted that 86% of patients had two or fewer relapses. Detailed yearly and other interim analyses of ARRs, EDSS, MRI changes (MRI cohort), and disability progression were also reported (see Gold et al. 2020) [93]. More detailed information regarding safety is also detailed in these analyses, especially regarding absolute lymphocyte count (ALC) and incidence of infection and malignancy (see above Safety, Adverse Events) [93].

### Safety, Adverse Events

The safety profile of monomethyl fumarate (MMF) comes largely from clinical trials of dimethyl fumarate (DMF) in MS patients, and the tolerability profile of MMF thus far comes from head-to-head comparison of MMF with DMF in healthy volunteers [94,95]. Common treatment-related adverse events in MS patients have included flushing, diarrhea, nausea, abdominal pain, vomiting, proteinuria, and pruritis [75,76]. Flushing and gastrointestinal events have been of mild or moderate severity, and were found to be highest in the first month of treatment by patient self-report [105]. Phase II trials also reported adverse events, including headache, fatigue, and feeling hot [86]. In phase III trials comparing DMF to glatiramer acetate (GA), no opportunistic infections or malignant neoplasms were reported, but there were decreased lymphocyte counts with DMF [75]. The issue of leukopenia and dimethyl fumarate-associated lymphopenia has since been investigated more closely [106,107]. In a cohort of 221 patients, 17% developed grade 2–3 lymphopenia, which did not resolve with DMF treatment, and smaller cohorts have shown similar results [106]. Patients over the age of 55 undergoing DMF treatment were found to be at increased risk of developing moderate to severe lymphopenia. In a 2-year prospective study of 456 MS patients treated with DMF, there were 95 cases of lymphopenia, with 13% grade 1, 7% grade 2, and 1% grade 3 [104]. A small number of cases of PML have been reported in MS patients taking DMF, including a patient without lymphocytopenia [87,108,109]. A small number of neoplasms were reported [110]. Clinically significant liver injury has been reported in more than 20 cases of MS patients treated with DMF [110,111]. FDA guidelines for Bafiertam™ (MMF) advise caution as opportunistic infections have occurred during DMF treatment, specifically herpes zoster, but also other viral, fungal, and bacterial pathogens [5]. Interim data analyses and follow-up on the long-term treatment continuation (~9 years) of the ENDORSE clinical trial (continuation of DEFINE and CONFIRM) appear to support a favorable risk-benefit profile of oral DMF [87,93]. Post-marketing data have noted DMF discontinuation due to lymphopenia and elevated transaminases, along with milder, transient reactions that did not result in discontinuation such as arthralgias, alopecia, and myalgias, and asymptomatic eosinophilia [110]. Among the pregnancies reported in the post-marketing setting and ongoing MS registries, there appears to be no increased risk of fetal abnormalities or adverse pregnancy outcomes [110,112,113].

Contraindications to MMF therapy include hypersensitivity (e.g., anaphylaxis or angioedema) to MMF, DMF, DRF, or any component of their formulations, or concomitant treatment with DMF or DRF, See Table 1 and Table 2.

## 6. Conclusions

MS is an autoimmune neurologic disorder affecting CNS myelin sheath and causing a plethora of symptoms, including pain, perception, sensory losses, autonomic dysfunction, gait disturbances, cognitive impairment, and psychiatric aberrancies. MMF is a novel oral agent that is FDA-approved for use in MS based on previous experience with DMF; the latter is metabolized into MMF, the active form. MMF works through activation of Nrf2 and NFkB to reduce oxidative stress and inflammation and alter the disease course in MS. It also reduces monocyte migration through the BBB through the downregulation of adhesion molecules, further reducing inflammatory effects. Other theories have been proposed, and the definitive mechanism is yet to be elucidated. MMF seems to be safe, well-tolerated, and effective to treat MS. Novel studies show that it may be better tolerated than DMF, though further studies are required to support this claim. Though no cure exists for MS, these novel therapies alter the course of the disease and provide patients with alternatives. More data are required to provide adequate conclusions about this drug treatment.

## Figures and Tables

**Table 1:** Clinical efficacy and safety.

Author (Year)	Groups Studied and Intervention	Results and Findings	Conclusions
Schimrigk et al. 2006 [97] Phase I	10 patients with definite RRMS, relapse in the year prior to enrollment, active lesion on MRI, active EDSS score 2.0–6.0; oral FAE * (Fumaderm initial^®^, Fumaderm forte^®^) 720 mg/day for 18 weeks, followed by 360 mg/day for 48 weeks	Reductions in mean number and total volume of gadolinium enhancing lesions (GdE) on T1 MRI after 18 weeks of treatment	Fumaric acid esters reduced radiologic progression of MS lesions in a small group of patients. Some FAE preparations contain more than 55% DMF and may be useful for RRMS patients.
Kappos et al. 2008 [86] Phase IIb	257 patients with RRMS; 120 mg of DMF QD, 120 mg of DMF TID, 240 mg of DMF TID, or placebo.	DMF 240 TID reduced mean number of GdE lesions (69%) over a 12 week period, number of new or enlarging T2-hyperintense lesions, new T1-hypointense lesions, and annual relapse rate (32%).	DMF can reduce radiologic progression of disease in RRMS patients. Consider DMF 240 mg TID for prevention of radiological progression of disease in RRMS patients.
Gold et al. 2012 [76] DEFINE Phase III	1234 RRMS patients; 240 mg DMF twice daily, 240 mg DMF thrice daily, or placebo.	Significantly lower estimated proportion of relapse: 27% in patients taking BID DMF, 26% taking TID DMF, and 46% with placebo. Annualized relapse rate at 2 years: 0.17 with BID, 0.19 with TID, and 0.36 with placebo. Rate of disability progression: 16% with BID, 18% with TID, and 27% with placebo. Significant reduction in number of Gd+ T2 MRI hyperintensities with each DMF regimen compared to placebo.	DMF regimens reduced MS relapses and imaging findings compared to placebo. Consider 240 mg DMF twice or thrice daily for MS patients unable to tolerate other MS treatments.
Malllucci et al. 2018 [114] Phase IV	Records of 720 MS patients (478 female) treated with DMF: 25.8% treatment-naïve; 19.5% discontinued another DMF treatment >12 months prior; 54.6% switched from another disease-modifying treatment (DMT): (IFN (45.8%), GA (27.2%), TFU (5.8%), FTY (7.3%), NTZ (6.6%). Median DMF exposure 17 months.	Reduction in ARR by 63.2% (mean ARR before DMF vs. mean ARR at least follow-up). 85% of patients relapse-free at 12 months, 76% of patients relapse-free at 24 months. 89% continued DMF at 12 months, and 70% continued DMF at 24 months.	DMF may be considered in patients who must switch from another disease-modifying therapy due to tolerance issues, lack of efficacy, or safety concerns.
Sabin et al. 2020 [110] Phase IV	886 MS patients (629 female) treated with DMF: 25.3% treatment-naïve; 74.7% switched from another DMT. Median exposure 39.5 months. 56.2% completed at least 3 years DMF treatment.	Tolerability and safety study. 71.2% experienced adverse events (flushing 44.1%, grade III lymphopenia 5.4%). 11.7% discontinued in the first year. No safety problems reported.	DMF may be considered a generally safe alternative to existing DMT for RRMS patients. Acknowledge that adverse effects are relatively common and there may occasionally be the need for discontinuation.
Gold et al. 2020 [93] ENDORSE Phase IV	1736 patients taking 240 mg of DMF who completed CONFIRM/DEFINE. Patients having taken GA or TID DMF excluded. Median follow-up 8.5 years (range 2.0–11.3).	ARR remained low (<0.20) over ~9 year treatment period. Approximately 70% patients had no new or enlarging MRI lesions compared to baseline after 7 years of DMF treatment. Of 2470 patients had ≥ lymphocyte assessment, 53 developed severe prolonged lymphopenia.	There is support for long-term safety and efficacy of DMF in RRMS patients.

* FAE: fumaric acid esters.

**Table 2:** Comparative studies.

Author (Year)	Groups Studied and Intervention	Results and Findings	Conclusions
Fox et al. 2012 [75] CONFIRM Phase III	1417 RRMS patients; DMF 240 mg BID, DMF 240 mg TID, glatiramer acetate subcutaneous 20 mg daily, or placebo.	Annualized relapse rate at 2 years: 0.22 with BID DMF, 0.20 with TID DMF, 0.29 with glatiramer acetate, and 0.40 with placebo. Significant reduction in number of new or enlarging T2 hyperintensities, and new T1 hypointensities. No significant reductions in disability progression comparing DMF regimens with glatiramer acetate.	Both DMF and glatiramer acetate reduced relapse rates and neuroradiologic progression of disease compared to placebo. No significant difference
Gold et al. 2017 [87] ENDORSE Phase IV	1736 patients who completed CONFIRM/DEFINE: All dose combinations represented (DMF BID and TID, placebo, and GA).	Cumulative ARR during 0–5 years for patients taking BID/BID was 0.163, versus patients taking placebo/BID 0.240. For the GA/BID patients, cumulative ARR was 0.199.	DMF treatment is associated with sustained low clinical disease activity and MRI progression. Treatment benefit may be sustained and safety profile may be favorable long-term.
Wehr et al. 2018 [96] Phase I	Direct pharmacokinetic comparison of monomethyl fumarate (MMF) and DMF. 35 healthy fasting volunteers, a single dose of 462 mg of MMF versus a single dose of 240 mg of DMF.	MMF was well-tolerated. Comparable mean concentrations of MMF and DMF over time. Adverse events 45.7% with MMF, and 54.3% with DMF.	The pharmacokinetic profiles of MMF and DMF are similar; the substances may be considered bioequivalent.
Naismith et al. 2020 [115] Phase III	504 patients with RRMS, randomized to diroximel fumarate (DRF) or DMF. BID 231 mg of DRF or BID 120 mg of DMF for 1 week; then BID 462 mg of DRF and BID 240 mg of DMF for 4 weeks. Tolerability and symptoms assessed by patient self-report.	Significantly reduction (46%) in Individual Gastrointestinal Symptom and Impact Scale (IGISIS) scores with DRF compared to DMF. Fewer gastrointestinal adverse events with DRF (34.8%) than with DMF (49.0%), and fewer discontinued DRF (1.6%) compared to DMF (5.6%).	DRF may have better short-term gastrointestinal tolerability than DMF.
Laplaud et al. 2019 [103] phase IV	1770 RRMS patients: 713 patients taking teriflunomide (TRF), 1057 taking DMF, evaluated at 2 years of treatment.	Adjusted proportion of patients with at least one new T2 lesion after 2 years of treatment, 60.8% for DMF, 72.2% for TRF. More patients were withdrawn from TRF (14.5%) than from DMF (8.5%) due to lack of effectiveness.	Class III evidence that TRF and DMF have similar clinical effectiveness for RRMS patients at 2 years. The larger patient population of this study may better reflect real-life MRI progression with DMF treatment.
